# Enhanced 2D Hand Pose Estimation for Gloved Medical Applications: A Preliminary Model

**DOI:** 10.3390/s24186005

**Published:** 2024-09-17

**Authors:** Adam W. Kiefer, Dominic Willoughby, Ryan P. MacPherson, Robert Hubal, Stephen F. Eckel

**Affiliations:** 1 Department of Exercise and Sport Science, University of North Carolina at Chapel Hill, Chapel Hill, NC 27599, USA; rpmac@email.unc.edu; 2 Human Movement Science Curriculum, University of North Carolina at Chapel Hill, Chapel Hill, NC 27599, USA; domwill@unc.edu; 3 Renaissance Computing Institute, University of North Carolina at Chapel Hill, Chapel Hill, NC 27599, USA; hubal@renci.org; 4 Eshelman School of Pharmacy, University of North Carolina at Chapel Hill, Chapel Hill, NC 27599, USA; sfeckel@email.unc.edu

**Keywords:** pose estimation, hand tracking, computer vision, machine learning, medical gloves, drug compounding, aseptic technique

## Abstract

(1) Background: As digital health technology evolves, the role of accurate medical-gloved hand tracking is becoming more important for the assessment and training of practitioners to reduce procedural errors in clinical settings. (2) Method: This study utilized computer vision for hand pose estimation to model skeletal hand movements during in situ aseptic drug compounding procedures. High-definition video cameras recorded hand movements while practitioners wore medical gloves of different colors. Hand poses were manually annotated, and machine learning models were developed and trained using the DeepLabCut interface via an 80/20 training/testing split. (3) Results: The developed model achieved an average root mean square error (RMSE) of 5.89 pixels across the training data set and 10.06 pixels across the test set. When excluding keypoints with a confidence value below 60%, the test set RMSE improved to 7.48 pixels, reflecting high accuracy in hand pose tracking. (4) Conclusions: The developed hand pose estimation model effectively tracks hand movements across both controlled and in situ drug compounding contexts, offering a first-of-its-kind medical glove hand tracking method. This model holds potential for enhancing clinical training and ensuring procedural safety, particularly in tasks requiring high precision such as drug compounding.

## 1. Introduction

As the field of digital health evolves, sensor technology is already playing a pivotal role in advancing critical assessments and the development of monitoring applications in medical diagnostics, treatment, safety, and training. The evolution of sensor technologies, including inertial measurement units, flex sensors, and computer vision, has opened new avenues for enhancing these capabilities while offering unprecedented precision and efficiency in behavioral and object tracking. Such technological advances offer promise for revolutionizing safety and training in clinical environments through enhanced performance tracking and monitoring systems, aiming to reduce the risk of procedural errors. 

One primary objective of these applications is to track performers’ hands to quantify both the overall motion trajectories and the specific kinematics of each hand and finger as tasks are performed. This tracking has been accomplished previously through sensors attached to the devices used in tasks, serving as proxies for hand trajectories [1], or with retroreflective markers in camera-based motion capture systems [2]. These methods have significantly enhanced insights into performance levels and have enabled objective measurements of skilled operations. For example, sensors have been used in healthcare-related studies to differentiate between the skills of expert and non-expert practitioners [2,3,4,5,6,7,8]. One such study used sensor-based accelerometry and retroreflective motion capture to reveal distinct differences in behavioral patterns between expert and novice providers during simulated laryngoscopy and intubation [5]. Electromagnetic sensors have also been used to index differences in motion parameters among experts and novices in separate studies involving simulated internal jugular cannulation [6], radiological “pin-pull” maneuvers [8], and large vessel patch anastomosis [3]. Furthermore, recent explorations into training applications have shown that virtual reality simulations, augmented with real-time hand tracking, enable surgeons to practice complex procedures safely, without the associated risks of live operations [9]. However, these techniques often face challenges when deployed in live clinical settings, especially with the sterile environment’s demand for non-invasive sensors compatible with medical gloves. Such limitations highlight the need for innovative approaches tailored to the unique demands of medical applications. 

Beyond enhancing surgical and medical procedure technique, sensor technologies also have the potential to play a crucial role in procedural safety, such as contamination prevention during drug compounding. For example, the 2012 meningitis outbreak caused by contaminated injections compounded at the New England Compounding Center (NECC) resulted in significant morbidity and mortality [10], highlighting the catastrophic consequences of inadequate procedural oversight and underscoring the inherent risks associated with compounding. This incident has led to stricter regulatory oversight and an increased demand for technological interventions to ensure patient safety. Advanced tracking technologies, particularly those capable of monitoring hand movements, are key to ensuring adherence to aseptic technique for preventing contamination [11]. To date, traditional monitoring methods, such as human observation or video recording, have failed to capture the intricate details of hand movements that can differentiate between proper and improper aseptic technique. There is likely significant potential for enhancing safety and compliance through greater precision in both the tracking and monitoring of these behaviors.

Despite the utility of sensor-based hand tracking across these various contexts, such methods are not without their limitations. For example, even with their small sizes, sensors often add weight or alter the shape of medical tools. Retroreflective markers, typically several millimeters in diameter and placed on medical devices or the individual’s hands, also introduce several challenges, including pre-procedure preparation time, potential contamination during the procedure leading to tracking errors, and added complexity to in situ medical assessments [4]. Alternatives, such as tracking systems integrated into gloves, may reduce the tactile feedback critical for precision movements and often limit tracking utility in simulation scenarios, detracting from the generalizability of the data collected. 

A recent letter to the editor emphasized the importance of motion-tracking technologies to enhance training and assessment in clinical settings, highlighting the necessity for methods that can accurately measure and analyze hand movements [12,13]. Advancements in computer vision technologies have introduced a compelling solution for clinical skill assessment and the training of complex medical procedures: hand pose estimation. This technique determines the position and orientation of the hands and their skeletal components through computational methods, marking a significant advancement from traditional motion capture technologies. This method operates without external markers or sensors, thereby allowing for the real-time capture of dynamic hand movements without adding burden to the performer. Specifically, by overcoming the challenges presented by earlier methods, hand pose estimation offers a tool for accurate modeling and analysis during in situ performance.

Hand pose estimation via computer vision and external cameras offers contactless, non-invasive communication between the human performer and the tracking technology through a variety of camera types and configurations [14]. These include the integration of additional sensor-based data such as red-green-blue (RGB) 2-dimensional (2D) video, time-of-flight sensors, and infrared sensors to extract information pertinent to hand configurations. Time-of-flight hand detection primarily uses depth sensors to provide 3-dimensional (3D) information without being affected by lighting or color changes. This enables the segmentation and recognition of regions of interest by analyzing depth fields and is especially useful in environments where visual information alone is not sufficient [15]. Other recognition techniques, such as those based on appearances or motion, focus on extracting features from image sequences to identify and analyze hand poses. Appearance-based methods rely on features from 2D images, such as pixel intensities, to model hand shapes and motions without prior segmentation [16]. This facilitates real-time processing and accommodates variance in user characteristics (e.g., medical glove color). Conversely, motion-based recognition tracks hand movements across video frames using algorithmic approaches. However, challenges such as lighting variations and dynamic backgrounds can reduce accuracy [17].

As technology has improved, alternatives to these approaches have become more viable. Specifically, skeleton-based recognition in hand pose estimation has shown promise over the last several years [18,19,20]. This method focuses on identifying model parameters that enhance the detection of complex hand features, including joint orientations, distances between joints, and the angles and trajectories of these joints. It leverages geometric attributes and translates features and correlations of data into a structured skeletal model of the hand. These types of models can effectively classify hand poses by examining the skeletal data that describe the relative positions of hand joints. Techniques used in skeleton-based recognition typically involve complex deep learning models, such as convolutional neural networks, to process the positional data of hand skeleton joints to accurately model the hand’s structure [19,20,21]. 

While advancements in skeleton-based hand pose estimation offer the potential to transform medical assessment and training applications, several challenges remain to be addressed. Current approaches struggle with achieving the accuracy and latency required for real-time or near real-time applications. Additionally, the sterile environment of clinical procedures imposes constraints, including the fact that performers typically wear a variety of different colored medical gloves, which challenge existing algorithms to accommodate. However, there has been notable work in related surgical domains. Hein et al. [22] proposed a hand pose estimation approach during surgical tool manipulation, utilizing a PVNet [23] model for object tracking in combination with the MANO hand model [24]. This hybrid solution was compared to both a HandObjectNet-based approach [25] and a hybrid PVNet–HandObjectNet model [23]. The MANO hand model, which captures low-dimensional hand shape variations rather than individual hand keypoints, contrasts with the HandObjectNet and PVNet–HandObjectNet approaches that integrate tool and hand pose together into a unified framework. Their evaluation indicated an average tool vertex error of 13.8 mm, though no specific hand pose error was reported. Similarly, Doughty et al. [26] developed a hybrid model, HMD-EgoPose, using an egocentric perspective via a Microsoft Hololens to track object-hand poses, and reduced the average tool’s 3D vertex error to 11.0 mm. 

While pose estimation techniques are increasingly used to track and assess medical task performance, they lack the precision needed for detailed hand kinematics analysis. One study explored hand kinematics in a medical context, employing a YOLOv5s [27] architecture combined with EfficientNet B3 and FPN-EfficientNet B1 modules [28]. Müller et al. [29] achieved a hand skeletal key point accuracy of 10.0 pixels, even with diverse medical glove colors, bloody gloves, and varied surgical tool manipulations. Though promising, this work relied on egocentric views, which are not always practical in situ. Notably, no current published algorithm effectively tracks multi-color gloved hand position in medical procedures from an exocentric perspective. Prior to the development of the current project, a search of existing exocentric perspective hand position models was conducted to identify an existing solution. The most well-known of these was MediaPipe Hands [30], a powerful computer-vision recognition tool that has been built to accurately recognize the position of hands. The long-term goal of our research team is to develop digital health applications for safety and protocol adherence, and this requires successful tracking of medical-gloved hands as part of the medical procedure workflow. Unfortunately, when we attempted to utilize MediaPipe Hands for gloved-hand tracking, it failed to detect any keypoints on gloved hands (i.e., it returned “NULL” values for all keypoints). This limitation has been noted in previous studies [28]. No other existing hand-tracking models are capable of tracking gloved hands from an exocentric perspective, which has necessitated an important advancement in the domain of medical procedure applications: the development of an exocentric hand-tracking model performant for medical-gloved hands of varying colors.

The purpose of the current manuscript is thus to examine a novel computer-vision based hand kinematics estimation method designed for use with gloved hands of various colors in medical settings as a crucial advancement for maintaining sterility while achieving high precision and efficiency. This method is designed to serve as a foundational component of a larger safety and adherence tracking platform for drug compounding, promising significant reductions in procedural errors and furthering enhancements in clinical training and safety protocols.

## 2. Materials and Methods

### 2.1. Data Collection

**Figure 1:**
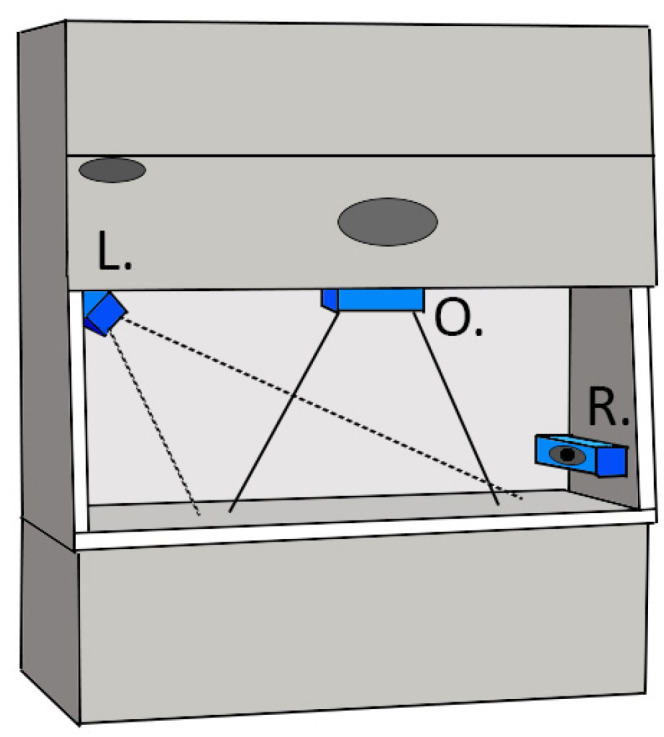
A schematic of the camera placement across both data collections of the in situ data collection. For the initial training data collection, the left (L) and overhead (O) camera views were used, while only the L camera was used for the in situ testing data collection. The right (R) camera was used for the initial training data collection; however, no frames were coded for inclusion in the training data set from this perspective.

Video data were collected in two separate locations. The first collection took place in a classroom laboratory at a major southeastern U.S. school of pharmacy, and videos were recorded on students practicing aseptic drug compounding procedures in a laminar airflow workstation (LAFW). For this collection, three commercially available Logitech BRIO webcams (Logitech, Lausanne, Switzerland) were affixed to the interior of the vent hood in strategic positions that limited airflow obstruction while optimizing hand visibility. All cameras were pointed at the center of the working area with perspectives from directly overhead (top-down), the upper left corner, and the right side of the hood (see Figure 1). During this collection, initial video recordings were taken of a student wearing latex gloves as they slowly rotated their hands on all axes to provide clear examples of hand position at a variety of angles. This was repeated twice for blue, purple, and tan medical glove colors for a total of six recordings. For the second collection, videos were recorded during a series of completed procedures in which each student completed two repetitions of compounding a drug using aseptic technique with all three glove colors for a total of six trials. Each session recording lasted roughly seven minutes and was recorded in 1080p resolution at 30 frames/second, resulting in approximately 25,000 frames of data per session. The second data collection was conducted in situ at the department of pharmacy of a major southeastern U.S. medical center. For this collection, only a single camera was placed in the upper left corner of the vent hood. A custom script automated both the start and stop of each recording once motion was detected in the video frame or once there was no longer any motion in the video frame, respectively. The camera was left in place for four days, and videos that did not contain a complete procedure from start to finish were discarded. A total of 237 videos were recorded, from which a subset of eight of these videos were selected, which included eight unique hand morphologies (eight separate people recorded) and eight distinct procedures. Each of the final eight videos ranged from four to nine minutes in length. Portions of these videos in which the subject stepped away from the hood and out of frame were manually removed.

**Figure 2:**
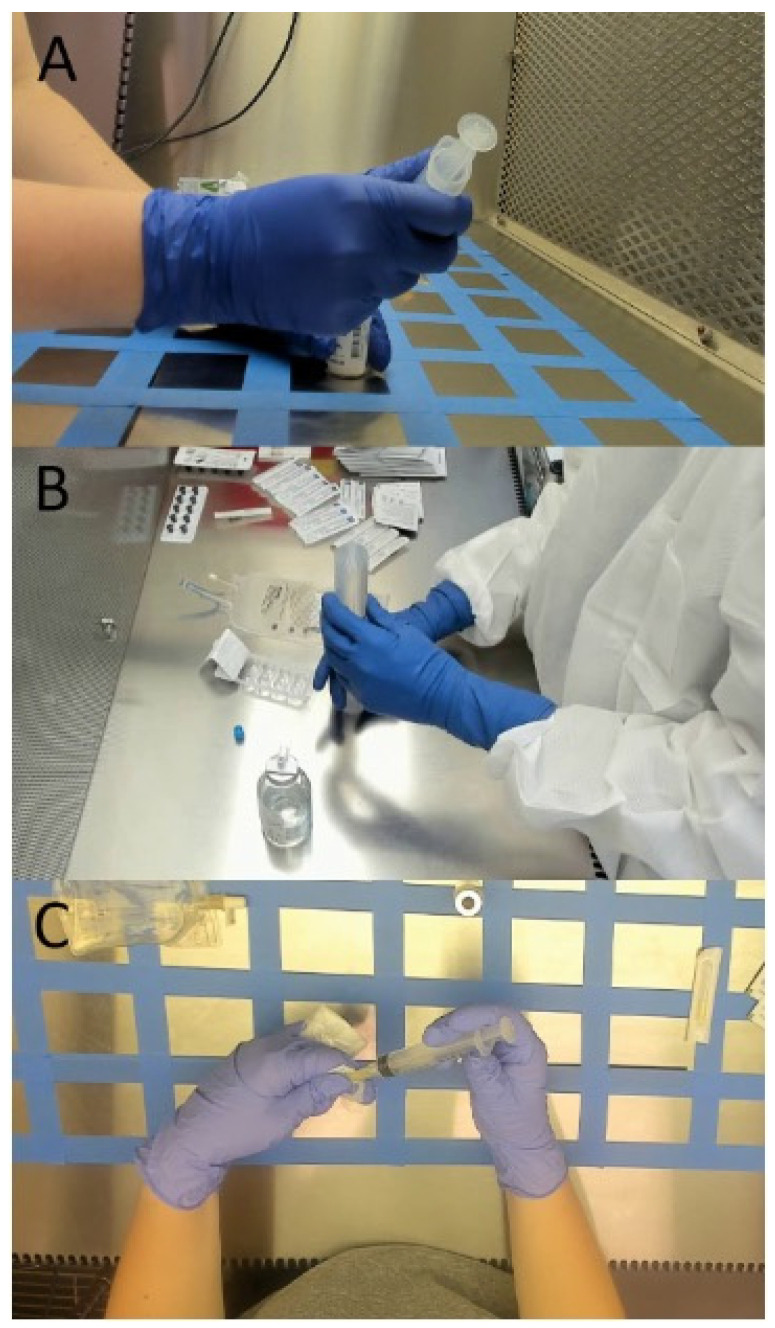
Comparisons between camera views: (**A**) camera position in the lower right corner of the LAFW, (**B**) camera position in the upper left of the LAFW, (**C**) overhead camera position within the LAFW.

The process of camera selection was based on two considerations: (1) visibility of each hand and (2) ease of application. The three camera positions shown in Figure 1 were assessed based on these two criteria. The overhead camera provided the clearest visibility of each hand in almost every circumstance, but the direction of airflow in the vent hoods could be disrupted whether it was a vertical or horizontal (i.e., LAFW) airflow hood. The lower right camera positioning was thus initially considered because it minimized airflow disruption; however, images from this camera were quickly discarded for poor visibility due to consistent occlusions. Figure 2 shows a still-frame image for all three camera perspectives. Figure 2A shows a typical image from the lower-right camera view, and it is important to note that the frequency at which the right hand blocked the entire view of the left hand severely limited the utility of this view for the purposes of the current project. Conversely, the balance of consistent visibility of both hands with a camera position that still minimally disrupted airflow was found in the upper left camera position (Figure 2B). In this position, the camera only blocked airflow on the extreme left side, which typically did not interrupt any use of the vent hood, while also capturing both left- and right-hand positions with minimal occlusion by other objects in the scene. The final camera position (Figure 2C) caused the greatest obstruction to airflow and was not considered a viable option for broader application.

### 2.2. Data Processing

**Figure 3:**
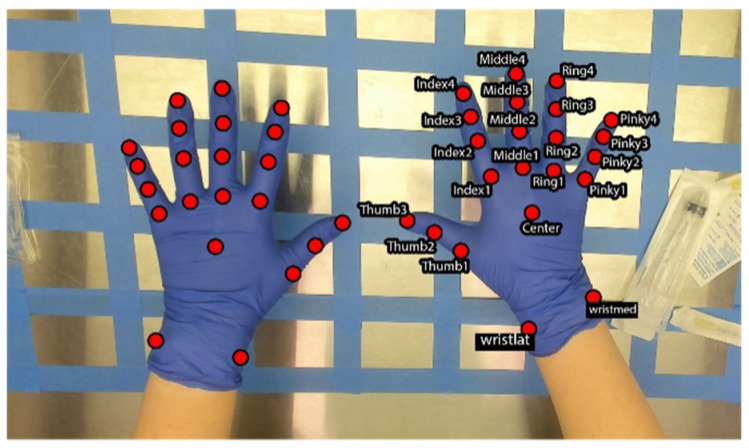
Twenty-two keypoints identified and labeled on the right hand. Labels are mirrored on the contralateral hand and follow an identical naming convention.

Video recordings were split into discrete images using a custom script (Python Ver. 3.7.11). A subset of approximately 700 images was saved from each video, equating to approximately 1 in every 12 images saved. This method allowed the images in the subset to be distinct from one another and representative of the behavior in the video without overwhelming human labelers, as markers are labeled manually by human coders. Images were then separated into groups of 50 and uploaded to Amazon Web Services (AWS, Amazon.com, Inc., Seattle, WA, USA). Images were labeled manually by research staff through the Amazon SageMaker Ground Truth data set labeling platform. Groups of 50 images were selected at a time for a single labeling job and sent to individual team members for labeling. In the labeling job setup, images were treated as video frames, and the task type of keypoints was selected. Twenty-two keypoints were identified for each hand, as shown in Figure 3. Markers included medial and lateral wrist markers (identified based on anatomical position), center of mass, and points on all metacarpophalangeal joints, interphalangeal joints, and fingertips, identified as 1–4 proximally to distally (1–3 for thumbs).

When onboarding new labelers, a set of 10 “gold standard” images was provided as an initial practice job to gain familiarity with the AWS platform, as well as to verify the accuracy of labeling across research staff. Upon completion of a labeling job, annotation data was compiled into a .json file that contained information on the x and y coordinate locations of each labeled point, as well as the identifications of each image labeled. This process ensured that recreations of the labels could be used to train future machine learning models. Of note, not every label could be identified in each image: labelers were instructed to only label keypoints they could clearly see and exclude keypoints that were occluded by other objects or other areas of the hand. The total count of annotated images was just under 1500. Given the number of workers available and the labor-intensive nature of this task, 1500 images represented an attainable balance of manageable workload and sufficient volume for this proof-of-concept model development.

### 2.3. Model Development

DeepLabCut is a computer vision machine learning program originally designed for tracking the position and movement of animals [31]. The program itself provides a full-service, start-to-finish user interface that allows individuals to take a series of video frames or individual images, annotate the desired keypoints, and train a machine learning model to automatically identify those keypoints on future unlabeled images. For the purposes of this project, AWS was utilized for marker identification due to its versatility and generalizability to other machine learning platforms. Deployment of this program was done through a Linux-based Ubuntu 20.04 EC2 environment on AWS.

Another custom Python script was developed to translate the AWS-generated .json annotation files into the appropriate file type for importing into the DeepLabCut Project Manager GUI 2.3.9. This script ultimately took individual .json files for individual images and collated them into a single monolithic .csv file that had a row for every image. The columns were categorized based on a series of four rows of metadata: “scorer”, “individuals”, “body parts”, and “coords”. The scorer row allowed research staff to track who labeled each image. The individuals row represented the individual instance of an object, which corresponded to the left and right hands, while the body parts row contained the names of each of the keypoints on a single hand. Finally, the coords row contained either an “x” or a “y” to indicate the x and y coordinates, respectively, for the keypoint identified by the metadata above. The cells in this monolithic .csv file, therefore, contained either the x or y coordinate in pixels for the image identified by the row for the keypoint identified by the metadata tags in the first 4 rows of that column. DeepLabCut has an in-built function to convert .csv files into .h5 files, which is the file type DeepLabCut GUI can read for information.

In the DeepLabCut GUI, projects are defined by a .yaml configuration file, which allows users to define keypoints, the skeleton model, individuals, and other similarly basic information. Once appropriate configurations are set, the monolithic .csv file is loaded into the GUI. Under the “Label Frames” tab, the “Check labels” button was used to verify that the annotations from the .json files were translated correctly and displayed correctly in the DeepLabCut GUI. Images were separated into standard 80% to 20% training/testing groups, and training was initiated.

DeepLabCut comes pre-loaded with three main machine learning architectures: ResNet, MobileNet, and EfficientNet. As the purpose of this project was to develop a model that balanced the requirements of being computationally lightweight with optimized accuracy in estimating hand position, we began by investigating MobileNet to maximize speed. In exchange for speed, MobileNet was not able to manage large images, nor could it provide high-resolution results on our data set. Based on MobileNet’s initial failure, it was determined that both MobileNet and EfficientNet would be unable to achieve usable accuracy without additional frame processing. Thus, a ResNet architecture became the primary focus. ResNet had the highest resolution, but at its most intricate instance of 152 layers, it took hours to complete a single batch of training, making it unsuited for developing a model that was fast to train without sacrificing accuracy. However, scaling back the complexity of the machine learning architecture allowed for greater speed. The architecture selected for this project was ResNet_50, a version of ResNet that has 50 discrete layers in the training structure. At this level of complexity, ResNet was able to provide high-resolution results in an acceptable timeframe, satisfying both criteria. Prior to the actual model training, this architecture allowed for some mild image pre-processing, which included removing color saturation and slightly boosting the contrast of each image. Training iterations were set to 50,000 and “checkpoints” were saved every 1000 iterations. Adding this layer of security allowed training to resume from any of those 50 save points in the case of technical issues (i.e., computer shutting down, program glitches, etc.). The learning rate scheduler was set to the DeepLabCut recommended defaults. Training took an average of four days to complete. Once training was complete, DeepLabCut provided a series of model snapshots and a .pickle file, which contained the final version of the model.

The evaluation tab on the DeepLabCut GUI allows the user to apply the model to a set of pre-labeled images that were not used to train the initial model. These images are presented without the labels, and the model generates its predictions independently. This process allows for a quantitative analysis of the accuracy of the model when compared to the pre-labeled keypoint locations, enabling assessment of model generalizability. Data produced by this process include an RMSE value on an individual key-point basis for each image, an overall summary RMSE value for all the validation images utilized, and test error in whole pixels with and without a confidence error cutoff. Prediction confidence percentage is a built-in value provided by the DeepLabCut model evaluation tools. For every point inferred by a provided model, DeepLabCut provides three output layers that account for vector field and intensity mapping: the x and y coordinates of a pixel and the calculated probability that the pixel defined by the previous two values is the location of the keypoint in question, on a scale of 0 to 1 [31]. The pixel location with the highest confidence value is reported; the x and y coordinates are used to generate inferred annotations, and the confidence value is reported for evaluation purposes. The prediction confidence cutoff was set to the default value of 60%.

## 3. Results

The final data set included 1483 images, 1186 (i.e., 80%) of which were used for the training process, and 297 (i.e., 20%) of which were used as a hold-out test set. After 50,000 training iterations, overall evaluation values were provided. Inferred values were compared to manually coded “ground truth” keypoint locations, and RMSE in absolute pixels was reported. Within the training set across all images and keypoints, there was a reported 5.89 pixel error. This indicates that, on average, the model-generated keypoint location was 5.89 pixels away from the human-coded location. When the model predicted keypoint locations for the test set, images that were not used as part of the training set, the RMSE error was reported at an average of 10.06 pixels. Reported pixel locations are also given as confidence values from 0 to 1. When points with a confidence value below 0.6 were excluded (i.e., points that were programmatically less than 60% confidence as identified correctly were not considered), the RMSE for the training set was 4.67 pixels, and the RMSE for the test set was 7.48 pixels. Table 1 lists average RMSE and confidence values for each keypoint across all 1483 images used in this data set. Notably, pixel error relates to metric error differently, depending on the distance of the object from the camera. In the present study, 1 pixel equates to 0.42 mm at the farthest distance. When considering the view in Figure 3, however, the hand is approximately 10 cm from the hood surface, which changes the conversion to 1 pixel equating to 0.54 mm. Using that reference image, the error was between 2.12 and 4.19 mm.

Importantly, a dependent *t*-test indicated no difference in RMSE for the left hand compared to the right hand when excluding keypoints with less than 60% confidence, *t*(21) = −1.934, *p* = 0.067 (M = 5.04 ± 1.202 and M = 5.364 ± 1.112 for the left- and right-hand keypoints, respectively). The left and right hands also exhibited a high average confidence score, with the left hand at an average of 95.1% and the right at an average of 91.9% (*p* = 0.008). Of particular note, the average RMSE values were inflated slightly due to specific keypoint markers underperforming. Specifically, the hand Center, Thumb 1, WristLat, and WristMed reported abnormally high RMSE values and lower average confidence values. On the left hand, those four markers were the only points to have less than 95% of the identified markers above the 60% confidence cutoff (hand Center: 94.55%, Thumb1: 94.79%, WristLat: 77.21%, WristMed: 88.35%) and had average confidence values below 95% (hand Center: 93%, Thumb1: 94%, WristLat: 77%, WristMed: 86%). The right hand had slightly different results. The markers with fewer than 95% of markers above a 60% confidence value were hand Center (74.39%), Middle1 (93.14%), Ring1 (83.21%), Ring2 (92.87%), Ring3 (94.09%), Pinky1 (82.89%), Pinky2 (90.18%), Pinky3 (93.77%), WristLat (83.15%), and WristMed (81.56%). Additionally, there were more markers with an average confidence value below 95% than on the left side: hand Center (77%), Index1 (94%), Middle1 (92%), Ring1 (84%), Ring2 (93%), Ring3 (93%), Pinky1 (83%), Pinky2 (90%), Pinky3 (93%), WristLat (82%), and WristMed (81%). When the four highlighted markers (hand Center, Thumb1, WristLat, WristMed) were factored out of average values, the average RMSE of points over 60% confidence dropped to 4.55 pixels on the left hand and 5.02 on the right hand.

## 4. Discussion

**Figure 4:**
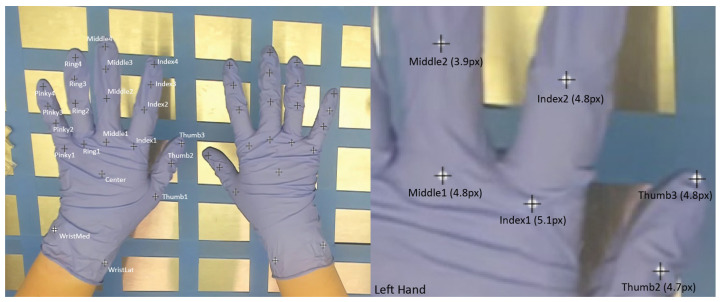
Representation of average inference error in pixels. The center of each black cross indicates the ground-truth marker location, while the white circle area indicates average RMSE in pixels. Note, while the image is magnified for visibility, the circles are to scale relative to an RMSE based on the full 1920 × 1080 pixel image.

The current study evaluated the accuracy of a first-of-its-kind hand pose estimation model for medical-gloved clinical applications using a combination of controlled and in situ data collections. The developed model performed well, as indicated by RMSE scores of less than 5.5 pixels, outperforming previously reported values by nearly 5 pixels [29]. Specifically, given the size of each image, an inferred point of 4–5 pixels from the ground-truth keypoint location provides a resolution of the hand that is accurate enough to predict keypoint positions within a drug-compounding task context. Figure 4 shows the RMSE error range, shown in white circles, around the ground-truth keypoint location as denoted by the black crosses. This image highlights that, when considering the average error, the keypoints are still highly representative of overall hand behavior. These results are additionally important given the inability to infer points on gloved hands using other models. Specifically, when these same images were input to MediaPipe Hands, the annotation file was returned empty, meaning that there was a 0% recognition rate. Our new model was capable of an average error of less than 5 pixels. This means that our model was able to recognize gloved hands with relatively high accuracy. This improvement shows a significant advancement in this domain. While this is an important first step, further empirical research is needed to determine whether this level of accuracy is appropriate for the practical needs of a given medical application.

**Figure 5:**
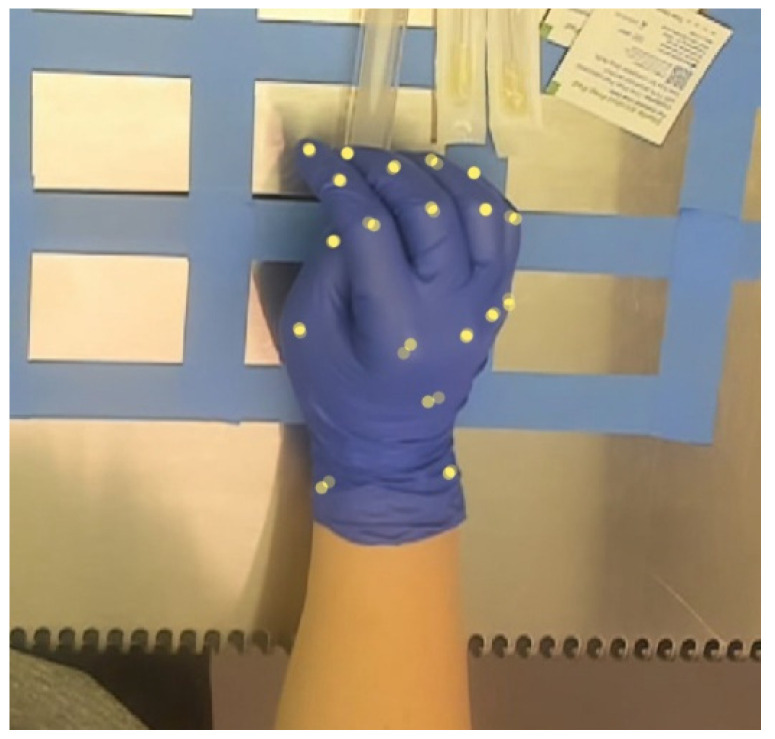
Error in manual labeling shown by overlaying two discrete images of the right hand from two different video frames, with the lighter and darker circles indicating independent labeling efforts of the same keypoint by the same human coder.

Also of importance is that of the 22 keypoints on each hand, only four had confidence scores that were notably worse than the others. These included the hand Center, Thumb1, WristLat, and WristMed markers, which all share a feature that reduces accurate identification: these keypoint locations do not always correspond to easy-to-identify morphological landmarks when gloves are worn. This makes it difficult for human coders to maintain reliability in position coding, which, in turn, leads to machine learning architectures training on less reliable data, resulting in less consistent labeling. The primary issue is that when a human coder manually labels featureless keypoints such as these, human coding error increases due to inconsistencies in accurately labeling the same pixel location within and between human labelers. This is unlike the remaining 18 keypoints, which map to specific, high-contrast anatomical landmarks that allow more accurate indexing of both manually labeled and inferred points. For example, Index4, the tip of the index finger, is easily identifiable, as are points related to the interphalangeal joints. This is clearly seen in each image, with higher-contrast landmark-based identifiers relative to those locations. When compared to the hand Center marker, whether on the palm or back of the hand and which represents the center of mass of the hand, these keypoints are situated on a smooth surface with a gloved hand. It is, therefore, very difficult to annotate a landmark-less location reliably, and Figure 5 highlights this issue. In this figure, two frames are taken from a video where the individual’s hand did not move between each frame, and these images have been overlayed to visualize the inconsistency of those keypoints. Manually labeling the same point without context provided by sequential images is challenging, and as shown in Figure 4, there was a small amount of error in this process. This error was likely exacerbated by the fact that sequential video frames may or may not have been presented sequentially to human coders. Additionally, up to 200 images were coded at one time, and thus attentional fatigue may have impacted the quality of some coding.

This issue, with certain keypoints having poorer confidence scores, has the potential to influence model development as well. For example, even if a machine learning model was given precise data, most machine learning models would struggle to identify a consistent spot on a featureless plane, especially with changing dimensions (e.g., different hands or different angles). Furthermore, when the challenges of labeling the ground-truth markers are passed on to the training data informing the development of a machine learning model, the model must attempt to find a set of rules for accurately identifying a challenging keypoint in the face of higher variability in input. This likely explains the marked reduction in performance for those specified markers.

It is also important to note that the model accuracy for the right hand was worse than the left hand. While the difference in average RMSE after the 60% cutoff did not show a statistically significant difference between the right and left hands (*p* = 0.067), there were more markers that performed below the 95% threshold compared to the left hand. On the left hand, the four aforementioned “trouble keypoints” were below the 95% threshold for both average confidence value and percentage of points. On the right hand, there were ten such markers, although for the remaining six that were not part of the four trouble keypoints, they were not far below the 95% threshold. The reduction in performance of the right hand may be explained by the position of the camera for most of the images provided for training. Figure 2B shows the image viewed by the camera when placed in the upper left corner of the LAFW. As the camera is on the left side of the volume, the left hand is far more visible and therefore easier to predict, while the right hand is occluded more frequently and simply further away from the camera, making it more difficult to infer points. Furthermore, the difference between the overhead image shown in Figure 2C and the upper left view for the left hand is much smaller than the change in shape of the right hand between those two camera views. Therefore, another potential explanation could be the inclusion of more disparate training images for the right hand and more similar images for the left hand. The overhead images were included because they provide the clearest image of both hands in a scene, but the upper left camera view was chosen as a fixed position that could remain consistent for both biological safety cabinet (vertical airflow) and LAFW (horizontal airflow) without blocking the required ventilation. This is an important consideration as a balance between model performance and the feasibility of video integration for in situ assessments. Furthermore, placing the camera high on the lateral wall gave the best view of the far hand, while placing the camera lower would risk occlusion of the near hand when objects get placed on that side of the vent hood. Regardless of these considerations, the average RMSE values for all points sans the four “trouble keypoints” were still within an acceptable range of error for the proposed aseptic technique task. An additional pixel of average error on the right hand still would not impede adequate detection of hand position, pose, and interaction with other objects in the scene during drug compounding. It remains to be seen whether this holds for other higher-precision medical tasks.

Despite these results, the study does face a few limitations that may impact the generalizability and application of these findings. First, as discussed, the variability in camera positioning may have introduced a bias in the hand visibility and likely affected the accuracy specific to right-hand pose estimation. This was potentially due to differences in both occlusion and distance from the camera. It also makes it difficult to project the model’s effectiveness across different camera setups while also indicating a potential issue in achieving uniform accuracy across both hands. Similarly, while manual labeling was necessary for the development of the model, reliance on this type of labeling can introduce human error. Therefore, future iterations of this project would benefit from a hybrid of more operationalized manual coding combined with the inclusion of calculated virtual markers for the trouble keypoints, as well as the repositioning of the camera to capture both the left and right hands equally. If a team of coders was deployed to label the keypoints manually for a new iteration, more careful guidelines for placement of markers on difficult areas of the hand (i.e., Center keypoints) would potentially improve the input to the model training and may result in a higher inference accuracy during model training. Finally, it would likely be beneficial in future iterations to remove the four poor-performing keypoints from manual coding entirely and use a heuristics-based approach to identifying those points after inference has been completed on the other, more stable markers. This would likely yield greater success and therefore lower average RMSE values. As it stands, while the resultant model is strong for applications such as drug compounding or relatively larger scale movements, such as during laryngoscopy and intubation, further examination is needed to determine the feasibility of the current model for more fine-grained movements such as those relevant to exacting surgical techniques.

In addition to the variations in shape, presentation, and visibility of hands, it is important to consider the color values of the gloves worn across tested images. The pre-processing of images done by our selected ResNet_50 architecture is insufficient to completely negate the effects of different colored gloves. Since the values of a light tan glove and a purple glove are different, removing hue saturation and slightly adjusting contrast does not cause the two different colors to appear similarly. Such an approach would require considerable distortion of the image, resulting in the contextual details that may play a role in hand detection being unrecognizable. Additionally, the amount of manual labor required to color match each image to one another would be considerable: in addition to labeling all 44 markers, coders would be required to adjust the contrast, saturation, brightness, and exposure of each image so that they fit a uniform value across all images. This would exponentially increase the required manual coding time. The simpler solution is to include an array of glove colors in the original training set to offset appearance differences between colors. Doing so will only slightly increase the number of images required for a reliable training set and does not require any additional steps outside the manual marker labeling already required.

**Figure 6:**
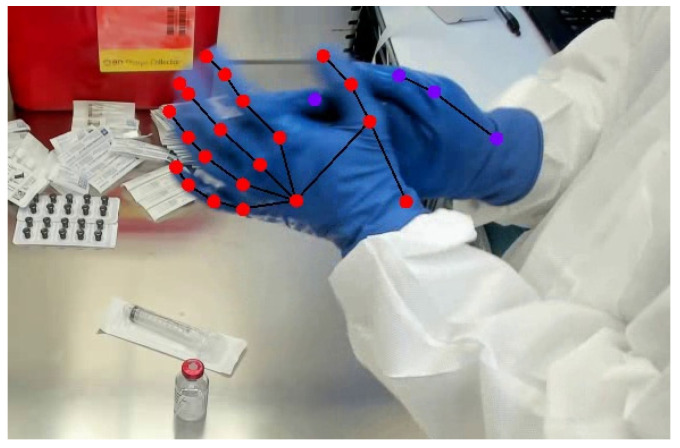
Video frame with annotations when the left hand occludes the right.

Despite these limitations, the development and successful implementation of an advanced hand pose estimation model, which is performant across different medical glove colors, directly addresses the challenges highlighted in the previous literature [17]. As shown in Figure 6 (see Supplemental Materials for complete video), the model we have developed is robust enough to provide accurate predictions even when there are occlusions present in the image. Moreover, this model has the potential to support technology that can revolutionize the way medical professionals train and successfully perform procedures by providing a more nuanced understanding of hand movements. This is crucial for tasks such as drug compounding and surgical operations, and the facilitation of more accurate and real-time tracking of hand poses in these settings will allow the resultant models to contribute to minimizing procedural errors and enhancing overall clinical safety. Ultimately, as computer vision-based tracking continues to evolve, the future of digital health will begin to take shape through the elucidation of new performance metrics and training applications, with hand tracking at the center of this important field.

## Figures and Tables

**Table 1:** RMSE and confidence percentage for all keypoints of the left and right hands.

Hand	Marker	Avg RMSE (pixels)	Avg Confidence (%)	Avg RMSE > 0.6 Confidence	%N > 0.6 Confidence	Hand	Marker	Avg RMSE (pixels)	Avg Confidence (%)	Avg RMSE > 0.6 Confidence	%N > 0.6 Confidence
Left	Center	7.480	0.93	6.289	94.55	Right	Center	13.114	0.77	6.667	74.39
	Index1	5.481	0.97	5.157	98.17		Index1	6.691	0.94	6.149	95.90
	Index2	5.070	0.98	4.802	98.80		Index2	5.665	0.98	4.796	98.30
	Index3	5.786	0.97	4.648	97.61		Index3	5.698	0.97	4.577	97.00
	Index4	5.102	0.96	4.440	96.63		Index4	5.281	0.97	4.249	96.47
	Middle1	5.206	0.98	4.776	98.39		Middle1	7.504	0.92	5.831	93.14
	Middle2	4.429	0.98	3.945	98.25		Middle2	5.583	0.95	4.629	95.95
	Middle3	5.649	0.95	4.923	96.54		Middle3	6.831	0.95	4.695	95.24
	Middle4	5.747	0.95	5.047	95.37		Middle4	6.820	0.96	4.352	95.24
	Ring1	4.553	0.97	4.308	97.67		Ring1	8.387	0.84	5.711	83.21
	Ring2	5.316	0.98	4.012	98.44		Ring2	7.059	0.93	4.820	92.87
	Ring3	5.166	0.95	4.731	96.15		Ring3	9.351	0.93	5.939	94.09
	Ring4	4.892	0.96	4.068	95.31		Ring4	8.113	0.95	4.323	95.56
	Pinky1	4.999	0.97	4.372	97.15		Pinky1	13.876	0.83	5.774	82.89
	Pinky2	4.325	0.97	4.165	97.47		Pinky2	9.020	0.90	4.719	90.18
	Pinky3	5.546	0.95	4.985	96.71		Pinky3	9.057	0.93	7.039	93.77
	Pinky4	5.893	0.96	4.072	97.08		Pinky4	5.812	0.95	4.158	95.52
	Thumb1	7.348	0.94	5.962	94.79		Thumb1	6.878	0.95	5.679	95.92
	Thumb2	5.046	0.98	4.693	98.52		Thumb2	4.837	0.98	4.568	98.80
	Thumb3	6.186	0.97	4.822	96.87		Thumb3	5.155	0.98	4.088	97.79
	WristLat	12.086	0.77	7.991	77.21		WristLat	9.784	0.82	7.724	83.15
	WristMed	11.346	0.86	8.559	88.35		WristMed	10.920	0.81	7.512	81.56

## Data Availability

Access to the data is restricted to safeguard proprietary information related to the trained models and their integration into future intellectual property claims. Data are available upon request and subject to permission for the sole purpose of peer review.

## Supplementary Materials

The following supporting information can be downloaded at: https://zenodo.org/records/11099114. Video S1: Side-by-side unlabeled and model-inferred keypoints of the preliminary medical-gloved hand pose model.
